# Prevalence and distribution of human papillomavirus genotypes among women attending gynecology clinics in northern Henan Province of China

**DOI:** 10.1186/s12985-021-01732-8

**Published:** 2022-01-06

**Authors:** Xiangpeng Wang, Yuan Song, Xiaofei Wei, Guanyu Wang, Ruili Sun, Mingyong Wang, Lijun Zhao

**Affiliations:** 1 Henan Key Laboratory of Immunology and Targeted Drugs, School of Laboratory Medicine, Xinxiang Medical University, Xinxiang, 453003 Henan China; 2 Department of Clinical Laboratory, Xinxiang Central Hospital, Xinxiang, 453000 Henan China

**Keywords:** Human papillomavirus, Genotype, Cervical cancer, Vaccine

## Abstract

**Background:**

Human papillomavirus (HPV) infection can cause cervical and other cancers, including vulva, vagina, penis, anus, or oropharynx. However, in China's northern Henan Province, data on the prevalence and genotype distribution of HPV among women attending gynecology clinics is limited. This study aimed to investigate the current prevalence and genotype distribution of HPV among women attending gynecology clinics in northern Henan Province.

**Methods:**

This study included 15,616 women aged 16–81 years old who visited the Xinxiang central hospital's gynecology department between January 2018 and December 2019. HPV DNA was detected by a conventional PCR method followed by HPV type-specific hybridization, which was designed to detect 17 high-risk HPV (HR-HPV) genotypes and 20 low-risk HPV (LR-HPV) genotypes. HPV prevalence and corresponding 95% confidence intervals (95% CI) were calculated using SPSS 18.0.

**Results:**

The overall HPV prevalence was 19.7% among women in northern Henan Province. Single, double, and multiple HPV infections accounted for 13.7%, 4.3%, and 1.8% of the total cases. Most infections were caused by HR-HPV (71.8%), and single genotype HPV infection (13.7%) was the most common pattern. The most common HR-HPV genotype was HPV16 (4.3%), followed by HPV52 (3.5%) and HPV58 (2.0%). The most common LR-HPV genotype was HPV6 (1.4%), followed by HPV61 (1.1%) and HPV81 (1.1%).

**Conclusions:**

HPV infection is high among women attending gynecology clinics in northern Henan Province. The highest prevalence was found in women less than 20 years old. In northern Henan Province, the 9-valent HPV vaccine is strongly recommended for regular immunization.

## Introduction

Cervical cancer, ranked after breast cancer, colorectal cancer, and lung cancer, is the fourth most common cancer among women worldwide, with approximately 530,000 new cases and 275,000 deaths every year [1–3]. According to current data, nearly 85% of women's deaths from cervical cancer occurred in developing or underdeveloped countries [1]. In China, cervical cancer is ranked as the eighth most common killer for women and continues to be a public health problem affecting women's health [4]. Human papillomavirus (HPV) is a sexually transmitted virus, which is mainly passed on through genital contact and also passed on by skin-to-skin contact. HPV infection can cause cervical cancer and other cancers, including the vulva, vagina, penis, anus, or oropharynx. Persistent infection with HR-HPV is the major cause of cervical cancer. Globally, HPV infection has been involved in more than 99% of cervical cancer [5]. HPV is a non-enveloped, double-stranded DNA virus with a genome of approximately 8.0 kb. As of 9 March 2015, more than 200 different types of HPV, identified numerically, have been identified by the International HPV Reference Center [6]. Based on epidemiological and biological data, twelve HPV types (16, 18, 31, 33, 35, 39, 45, 51, 52, 56, 58 and 59) have been classified as HR-HPV [7]. Eight HPV types (26, 53, 66, 67, 68, 70, 73 and 82), identified as single HPV infection in about 3% cervical cancer, are classified as probable/possible (p) HR-HPV due to lack of biological data [8]. It is extremely difficult to isolate and culture HPV in vitro. Additionally, not all patients infected with HPV have an obvious antibody response. Thus, HPV DNA detection by PCR becomes a non-invasive and sensitive method for confirming cervical HPV infection. Since persistent infection with HR-HPV is a necessary cause for cervical cancer development, DNA detection and genotyping of HPV can be an essential method for controlling and preventing HPV-related diseases in China.

Up to present, three licensed prophylactic vaccines, a bivalent vaccine against HPV16 and 18, a quadrivalent vaccine against HPV 6, 11, 16, and 18, and a 9-valent vaccine against HPV 6, 11, 16, 18, 31, 33, 45, 52 and 58, are considered as effective and safe for preventing HPV infection. However, the current vaccines offer protection only targeted at a few HPV genotypes and provide cross-protection for certain HPV genotypes. For example, the bivalent vaccine against HPV16 and 18 has a cross-protection against HPV 31, 34 and 45 [9]. The vaccines cannot prevent infection of all subtypes of HPV. Since the prevalence of HPV infection in women shows geographical distribution [10, 11], understanding HPV prevalence would be necessary to introduce an HPV vaccination program for cervical cancer prevention.

At present, the three vaccines against HPV can be used in China. However, only the bivalent vaccine and the quadrivalent vaccine against HPV are available in northern Henan Province. The 9-valent vaccines are only used in some provinces in China since May 2018. Lack of epidemiological data on HPV infection and genotype distribution makes it difficult to implement preventative measures such as HPV vaccination in northern Henan Province. The sponsors and local government may support HPV vaccines by using the scientific data of HPV prevalence. Therefore, the prevalence and genotype distribution of HPV among women attending gynecology clinics in Henan Province were identified in this study. The results of this research are of great importance for estimation of the awareness of HPV infection and the introduction of vaccination program in northern Henan Province of China.

## Materials and methods

### Study population and specimen collection

Women aged 16 and 81 attending regular gynecological outpatient clinics in Xinxiang central hospital between January 2018 and December 2019 in northern Henan Province were invited to participate in this study. They were divided into six age groups. The G1 group meant age < 20; the G2 group meant 20 ≤ age < 30; the G3 group meant 30 ≤ age < 40; the G4 group meant 40 ≤ age < 50; the G5 group meant 50 ≤ age < 60; the G6 group meant age ≥ 60. Women were excluded for: the presence of cervical cancer, pregnancy at the time of enrollment, previous HPV vaccination, without age information, hysterectomy, or immunosuppression. Finally, a total of 15,616 participants were included in this study for analysis.

Cervical specimens were collected by the trained clinicians assisted by the speculum in Xinxiang central hospital. The endocervical and ectocervical cells were collected from the cervical canal using a plastic brush (Hybribio limited Corp, Chaozhou, Guangdong, China). The brush was placed into a 2 mL vial of Hybribio cervical cells preservation solution (Hybribio limited Corp, Chaozhou, Guangdong, China) for HPV DNA detection. This study was performed strictly following the Declaration of Helsinki and approved by the Ethics Committee in Xinxiang Medical University. Informed consent was obtained from all participants before enrollment.

### DNA extraction, PCR amplification, and HPV genotyping

HPV DNA was extracted from the cervical cells using the Hybribio viral DNA extraction kit (Hybribio limited Corp, Chaozhou, Guangdong, China). Briefly, the cervical cells were first digested by proteinase K. Then, the released DNA was obtained through absorption to magnetic glass particles, washed, and purified from these particles using the automated nucleic acid extraction instrument (Hybribio limited Corp, Chaozhou, Guangdong, China). The concentration and purity (OD260/OD280 1.6–1.8) of DNA were determined by Nanodrop 2000 (Thermo Fisher Scientific, CA, USA). After the concentration was determined, the samples were stored.

HPV DNA amplification and genotyping were conducted using a commercial Hybribio HPV genotyping detection kit (Hybribio limited Corp, Chaozhou, Guangdong, China) for 37 HPV types, including 17 HR-HPV types (16, 18, 26, 31, 33, 35, 39, 45, 51, 52, 53, 56, 58, 59, 66, 68, 82) and 20 LR-HPV types (6, 11, 34, 40, 42, 43, 44, 54, 55, 57, 61, 67, 69, 70, 71, 72, 73, 81, 83, 84). The China Food and Drug Administration has authorized the kit for clinical use. The HPV L1 consensus biotinylated primer sets were used in the PCR assay. 1 μL of the DNA was used in the 25 μL PCR master mix. PCR reaction was initiated with denaturation at 95 °C for 9 min, followed by 40 cycles of denaturation at 95 °C for 20 s, annealing at 55 °C for 30 s and elongation at 72 °C for 30 s, with a final extension at 72 °C for 5 min.

HPV genotyping was performed by the flow-through hybridization method. The flow-through hybridization was performed on a medical nucleic acid hybridization instrument prewarmed at 45 °C prior to usage. A nylon membrane on which 37 HPV genotype-specific oligonucleotide probes secured was placed into the instrument. The biotinylated PCR product was denatured at 95 °C for 5 min and then chilled on ice for 2 min before hybridization. The PCR product was mixed with the hybridization solutions, and the mixture was added into the sample wells to conduct flow-through hybridization for 10 min. The nylon membrane was washed with the hybridization solution three times, and the empty region was blocked without reaction. The hybridizing signal was detected with streptavidin alkaline phosphatase, binding to biotinylated PCR products, and its subtract NBT/BCIP (nitro-blue tetrazolium-5-Bromo-4-chloro-3-indolyl phosphate). The genotyping result was detected by the position of the HPV-genotype probes on the membrane. The blue dot on the membrane, indicating a positive result, was judged by the naked eyes. The experimental procedures, including PCR and flow-through hybridization, followed the manufacturer's manual. Multiple dots showed multiple infections. Quality controls were carried out throughout the experiment, including PCR amplification and hybridization by using positive and negative controls provided by the kit.

### Statistical analysis

HPV prevalence and genotype distribution were analyzed. Single, double, and multiple HPV infections were defined as infections with one, two, and more than two genotypes of HPV infections. HPV prevalence in designated groups and corresponding 95% confidence intervals (95% CI) were calculated using SPSS 18.0 for Windows (SPSS Inc., IL, USA).

## Results

### Overall and age-specific HPV prevalence

From January 2018 to December 2019, 15,616 cervical specimens collected for HPV DNA detection were used for statistical analysis. There were 3081 specimens positive for any HPV DNA, and the overall prevalence of HPV was 19.7% (95% CI 19.1–20.4%). The 15,616 participants were divided into six age groups, and the HPV infection rate in each group was calculated as shown in Table 1. HPV infection was distributed in each age group with the infection rates from 17.7 to 41.8%. The highest prevalence of HPV infection was found among women in the G1 group with an infection rate of 41.8% (95% CI 36.5–47.2%), followed by an infection rate of 22.9% (95% CI 20.2–25.5%) in the G6 group and an infection rate of 21.1% (95% CI 19.6–25.5%) in the G2 group.

**Table 1:** The prevalence of HPV among women in different groups

Group	Age, y	Sample size	Positive no	% (95% CI)
G1	< 20	325	136	41.8 (36.5–47.2)
G2	20–29	2874	605	21.1 (19.6–22.5)
G3	30–39	4222	765	18.1 (17.0–19.3)
G4	40–49	4829	856	17.7 (16.6–18.8)
G5	50–59	2417	502	20.8 (19.2–22.4)
G6	> 60	949	217	22.9 (20.2–25.5)
Total		15,616	3081	19.7 (19.1–20.4)

### Distribution of single, double, and multiple HPV infections

Single genotype HPV infection (13.7%) was the most common pattern, and it occurred more frequently than double (4.3%) and multiple HPV infections (1.8%) shown in Table 2. The group with the highest infection rate was the G1 group (28.0%, 95% CI 36.5–47.2%). The infection rates declined as the age increased in the G6 group. Double infections had a high infection rate in G1 group at 10.8% and were also increasing with sharp decrease in G3 group. For multiple infections, the highest infection rate was in the G1 group (3.1%) and the lowest in the G4 group (1.3%).

**Table 2:** Single, double, and multiple HPV infections in different groups

Group	Age, y	Sample size	Single infection		Double infections		Multiple infections	
			Positive no	% (95% CI)	Positive no	% (95% CI)	Positive no	% (95% CI)
G1	< 20	325	91	28.0 (23.1–32.9)	35	10.8 (7.4–14.2)	10	3.1 (1.2–5.0)
G2	20–29	2874	396	13.8 (12.5–15.0)	151	5.3 (4.4–6.1)	58	2.0 (1.5–2.5)
G3	30–39	4222	562	13.3 (12.3–14.3)	142	3.4 (2.8–3.9)	61	1.4 (1.1–1.8)
G4	40–49	4829	611	12.7 (11.7–13.6)	181	3.7 (3.2–4.3)	64	1.3 (1.0–1.6)
G5	50–59	2417	331	13.7 (12.3–15.1)	115	4.8 (3.9–5.6)	56	2.3 (1.7–2.9)
G6	> 60	949	143	15.1 (12.8–17.3)	43	4.5 (3.2–5.9)	31	3.3 (2.1–4.4)
Total		15,616	2134	13.7 (13.1–14.2)	667	4.3 (4.0–4.6)	280	1.8 (1.6–2.0)

### HPV genotype distribution

There were 36 different HPV genotypes, including 17 HR-HPV genotypes and 19 LR-HPV genotypes, identified in this study. The prevalence of 17 HR-HPV was demonstrated in Table 3. The most common HR-HPV identified was HPV16 (4.3%), followed by HPV52 (3.5%), HPV58 (2.0%), HPV53 (1.8%) and HPV39 (1.5%). To be noted, HPV18 was only the seventh most common HR-HPV genotype to be detected. The top five genotypes for individuals with a single HR-HPV infection were HPV16, HPV52, HPV58, HPV53, and HPV39. For individuals with double HPV infections, the HR-HPV that ranked top five were HPV16, HPV58, HPV52, HPV39, and HPV53. For individuals with multiple HPV infections, the HR-HPV that ranked top five were HPV52, HPV16, HPV58, HPV51, and HPV53. These data suggested that HPV16 infection was predominant in HPV-positive patients. The prevalence of 19 LR-HPV genotypes was demonstrated in Table 4. The most common LR-HPV identified was HPV6, followed by HPV61, HPV81, HPV54, and HPV11. The most commonly detected genotype for individuals with a single LR-HPV infection was HPV61, followed by HPV54, HPV6, HPV81, and HPV11. For double and multiple HPV-infected individuals, HPV6 and HPV61 were the two most common LR-HPV. The prevalent genotypes ranked top 10 were HPV16, HPV52, HPV58, HPV53, HPV39, HPV51, HPV6, HPV18, HPV61, and HPV81. HR-HPV infection accounted for 71.8%, demonstrating the most infection was caused by HR-HPV in northern Henan Province.

**Table 3:** Distribution of HR-HPV genotypes in study participants

HPV type	Single infection		Double infections		Multiple infections		Total infections
	Positive no	% (95% CI)	Positive no	% (95% CI)	Positive no	% (95% CI)	% (95% CI)
HPV16	380	2.4 (2.2–2.7)	184	1.2 (1.0–1.3)	105	0.7 (0.5–0.8)	4.3 (4.0–4.6)
HPV52	185	1.2 (1.0–1.4)	98	0.6 (0.5–0.8)	269	1.7 (1.5–1.9)	3.5 (3.2–3.8)
HPV58	144	0.9 (0.8–1.1)	106	0.7 (0.5–0.8)	61	0.4 (0.3–0.5)	2.0 (1.8–2.2)
HPV53	138	0.9 (0.7–1.0)	81	0.5 (0.4–0.6)	59	0.4 (0.3–0.5)	1.8 (1.6–2.0)
HPV39	95	0.6 (0.5–0.7)	90	0.6 (0.5–0.7)	45	0.3 (0.2–0.4)	1.5 (1.3–1.7)
HPV51	88	0.6 (0.4–0.7)	69	0.4 (0.3–0.5)	61	0.4 (0.3–0.5)	1.4 (1.2–1.6)
HPV18	96	0.6 (0.5–0.7)	32	0.2 (0.1–0.3)	40	0.3 (0.2–0.3)	1.1 (0.9–1.2)
HPV33	74	0.5 (0.4–0.6)	53	0.3 (0.2–0.4)	32	0.2 (0.1–0.3)	1.0 (0.9–1.2)
HPV31	70	0.4 (0.3–0.6)	46	0.3 (0.2–0.4)	29	0.2 (0.1–0.3)	0.9 (0.8–1.1)
HPV68	57	0.4 (0.3–0.5)	38	0.2 (0.2–0.3)	27	0.2 (0.1–0.2)	0.8 (0.6–0.9)
HPV66	55	0.4 (0.3–0.4)	31	0.2 (0.1–0.3)	33	0.2 (0.1–0.3)	0.8 (0.6–0.9)
HPV56	43	0.3 (0.2–0.4)	33	0.2 (0.1–0.3)	35	0.2 (0.1–0.3)	0.7 (0.6–0.8)
HPV59	27	0.2 (0.1–0.2)	20	0.1 (0.1–0.2)	23	0.1 (0.1–0.2)	0.4 (0.3–0.6)
HPV45	38	0.2 (0.2–0.3)	10	0.1 (0.0–0.1)	10	0.1 (0.0–0.1)	0.4 (0.3–0.5)
HPV35	18	0.1 (0.1–0.2)	10	0.1 (0.0–0.1)	12	0.1 (0.0–0.1)	0.3 (0.2–0.3)
HPV82	14	0.1 (0.0–0.1)	15	0.1 (0.0–0.1)	8	0.1 (0.0–0.1)	0.2 (0.2–0.3)
HPV26	15	0.1 (0.0–0.1)	13	0.1 (0.0–0.1)	5	0.0 (0.0–0.1)	0.2 (0.1–0.3)

**Table 4:** Distribution of LR-HPV genotypes in study participants

HPV type	Single infection		Double infections		Multiple infections		Total infections
	Positive no	% (95% CI)	Positive no	% (95% CI)	Positive no	% (95% CI)	% (95% CI)
HPV6	78	0.5 (0.4–0.6)	88	0.6 (0.4–0.7)	52	0.3 (0.2–0.4)	1.4 (1.2–1.6)
HPV61	111	0.7 (0.6–0.8)	27	0.2 (0.1–0.2)	30	0.2 (0.1–0.3)	1.1 (0.9–1.2)
HPV81	70	0.4 (0.3–0.6)	55	0.4 (0.3–0.4)	43	0.3 (0.2–0.4)	1.1 (0.9–1.2)
HPV54	79	0.5 (0.4–0.6)	51	0.3 (0.2–0.4)	32	0.2 (0.1–0.3)	1.0 (0.9–1.2)
HPV11	49	0.3 (0.2–0.4)	35	0.2 (0.1–0.3)	32	0.2 (0.1–0.3)	0.7 (0.6–0.9)
HPV40	48	0.3 (0.2–0.4)	23	0.1 (0.1–0.2)	9	0.1 (0.0–0.1)	0.5 (0.4–0.6)
HPV84	27	0.2 (0.1–0.2)	26	0.2 (0.1–0.2)	25	0.2 (0.1–0.2)	0.5 (0.4–0.6)
HPV34	27	0.2 (0.1–0.2)	14	0.1 (0.0–0.1)	15	0.1 (0.0–0.1)	0.4 (0.3–0.5)
HPV70	16	0.1 (0.1–0.2)	16	0.1 (0.1–0.2)	10	0.1 (0.0–0.1)	0.3 (0.2–0.4)
HPV44	20	0.1 (0.1–0.2)	12	0.1 (0.0–0.1)	9	0.1 (0.0–0.1)	0.3 (0.2–0.3)
HPV42	21	0.1 (0.1–0.2)	9	0.1 (0.0–0.1)	8	0.1 (0.0–0.1)	0.2 (0.2–0.3)
HPV55	10	0.1 (0.0–0.1)	16	0.1 (0.1–0.2)	3	0.0 (0.0–0.0)	0.2 (0.1–0.3)
HPV73	12	0.1 (0.0–0.1)	5	0.0 (0.0–0.1)	11	0.1 (0.0–0.1)	0.2 (0.1–0.2)
HPV43	4	0.0 (0.0–0.1)	8	0.1 (0.0–0.1)	9	0.1 (0.0–0.1)	0.1 (0.1–0.2)
HPV69	8	0.1 (0.0–0.1)	6	0.0 (0.0–0.1)	4	0.0 (0.0–0.1)	0.1 (0.1–0.2)
HPV57	4	0.0 (0.0–0.1)	9	0.1 (0.0–0.1)	0	0.0 (0.0–0.0)	0.1 (0.0–0.1)
HPV83	5	0.0 (0.0–0.1)	3	0.0 (0.0–0.0)	3	0.0 (0.0–0.0)	0.1 (0.0–0.1)
HPV67	3	0.0 (0.0–0.0)	1	0.0 (0.0–0.0)	4	0.0 (0.0–0.1)	0.1 (0.0–0.1)
HPV71	5	0.0 (0.0–0.1)	1	0.0 (0.0–0.0)	2	0.0 (0.0–0.0)	0.1 (0.0–0.1)

## Discussion

China has a vast territory and a large population of more than 1.4 billion, and the prevalence of HPV varies greatly in different provinces of China. Some studies reported the prevalence and genotype distribution of HPV in different provinces in China [12–15]. However, there are few reports on HPV prevalence in Henan Province. The present study provided the prevalence and genotype data of HPV from women attending regular gynecological outpatient clinics from January 2018 to December 2019 in the northern Henan Province of China. Our study showed that the overall HPV infection rate was 19.7%, which was similar to the results from several surveys of HPV prevalence reported in other provinces of China [16–18]. The prevalence of HPV in northern Henan Province was lower than that reported in Shandong Province (28.4%) and Fujian Province (38.3%) [19, 20], but higher than Yunnan Province (12.9%) and Shanxi Province (8.92%) [12, 21]. The different HPV prevalence in northern Henan Province was expected and could have been due to the different economic conditions, living habits and customs, cultural diversity, sampling strategy, and the HPV detection methods [13, 22]. According to the previous reports, the HPV infection rate ranges from 6.7 to 44.5% in China [23]. Although the HPV infection rates in northern Henan Province are not the highest in China, the women need education on prevention and control of HPV infection and the related diseases.

This present study provided age-specific HPV infection rates among women. Our results showed that women under 20 years had the highest infection rate. Single, double and multiple infections rates were also the highest in women under 20 years. There are two possible reasons for this phenomenon: the first reason is that literature shows that HPV prevalence is higher in the younger age group [22, 24]. The second reason is that younger women may engage in unprotected sexual activities, exposing them to infections. The above two reasons led to the highest infection rate in the G1 group. It was reported that young women infected with HPV were temporary, and the immune system would clear the virus in most cases [25, 26]. Therefore, the infection rate of HPV will gradually decline with age increased.

The present study found that the prevalence of HPV declined gradually in the middle-aged groups and slightly increased in the G6 group (22.9%), which indicated that women over 60 years suffered severely from HPV infection. For women over 60 years, HPV infections may occur when they are young, and the infections probably are persistent infections instead of new HPV infections. If the HPV cannot be eliminated by the immune system and persist for several years, this will present as a risk factor for neoplasia development. HPV infection rates was 21.1% in women aged 20–29, 18.1% in women aged 30–39, 17.7% in women aged 40–49 and 20.8% in women aged 50–59. According to recent data from China National Cancer Center, there were 98,990 new cases of cervical cancer and 30,500 deaths in China in 2015 [27]. The number of new cervical cancer cases was 10,948 in Henan Province, with an incidence of 21.09 per 100,000 [28]. Since HPV infection is a major etiological factor for cervical cancer development, future treatment that could eliminate HPV infection and prevent the progression of cervical cancer is of great importance.

Our present study also provided the age-specific distribution of single, double, and multiple HPV infections rates. Except for the G1 group, the single, double, and multiple HPV infections rates of the rest groups were very close. There has not been a consensus on whether numerous HPV infections raise the risk of cervical cancer more than a single HPV infection up until now. Some studies have reported that multiple HPV infections had a higher risk of cervical cancer occurrence and development than single HPV infections [29, 30]. However, other studies have found that single HPV infections had a greater risk of developing cervical cancer concerning multiple infections [31, 32]. In our study, the prevalence of single HPV infections is higher than that of double and multiple HPV infections, accounting for 13.7% of the total cases, and HPV16 was the most commonly detected genotype in a single HPV infection. HPV16 infection is the one with the highest oncogenic risk [32]. Thus, more attention should be paid to single HPV16 infection. The double HPV infections and multiple HPV infections accounted for 4.3% and 1.8% of the total cases, respectively. The investigation of double and multiple HPV infections is of great importance to study the prevalence of HPV and is also of great significance to develop a multivalent HPV vaccine.

It is important to know the HPV genotypes distribution since data concerning the distribution of HPV genotypes is concerning with the vaccine development. Persistent infection with HR-HPV is the primary etiological factor for cervical cancer. Up to 70% of cervical cancers are caused by HPV16 and HPV18, with the remainder of cervical cancers caused by other HR-HPV genotypes. In the present study, HPV16, HPV52, HPV58, HPV53, and HPV39 were the top five HR-HPV genotypes. HPV16 ranked first in our study, and the infection rate reached up to 4.3%. HPV52 and HPV58 ranked second and third, respectively. Besides HPV16, both HPV52 and HPV58 are also strongly correlated with cervical cancer development [31]. The top three genotypes distribution pattern is consistent with the data from several previous Chinese population-based HPV investigations [19, 33, 34]. It was reported the HPV18 ranked second in Chinese women between 1991 and 2016 [4]. In our study, the HPV18, with an infection rate of 1.1%, ranked seven in the HR-HPV genotypes, which was consistent with the recent results in several other regions in China [19, 21, 22].

Furthermore, the study also investigated the distribution of LR-HPV. Our study showed that the top five most common LR-HPV genotypes were HPV6, HPV61, HPV81, HPV54, and HPV11. For the LR-HPV types, cervical cancer association is very rare in the general women population. LR-HPV mainly causes genital warts, common warts, flats warts, and many other skin lesions [35], which are usually asymptomatic but are sometimes accompanied by inching, burning, or bleeding, leading to psychosocial disturbances. The 9-valent HPV vaccine is used to prevent infection with HPV6, HPV11, HPV16, HPV18, HPV31, HPV33, HPV45, HPV52, and HPV58. Our results support the recommendation of the 9-valent HPV vaccine for routine vaccination in northern Henan Province. Meanwhile, vaccines against HPV53, HPV39, and HPV51 should also be developed for women in this area.

## Conclusions

A high prevalence of HPV was reported in the study. HPV16, HPV52, and HPV58 were the dominant HR-HPV in northern Henan Province. The results in our study provide important information for cervical cancer screening and vaccination in women in northern Henan Province of China.

## Data Availability

The data collected from Xinxiang central hospital in Xinxiang city can be freely shared. Any additional information may be obtained from the corresponding author on a reasonable request.

## Abbreviations

HPV: Human papillomavirus
HR-HPV: High-risk HPV
LR-HPV: Low-risk HPV
95% CI: 95% Confidence intervals
